# Joint Genomic Prediction of Canine Hip Dysplasia in UK and US Labrador Retrievers

**DOI:** 10.3389/fgene.2018.00101

**Published:** 2018-03-28

**Authors:** Stefan M. Edwards, John A. Woolliams, John M. Hickey, Sarah C. Blott, Dylan N. Clements, Enrique Sánchez-Molano, Rory J. Todhunter, Pamela Wiener

**Affiliations:** ^1^The Roslin Institute and Royal (Dick) School of Veterinary Studies, The University of Edinburgh, Midlothian, United Kingdom; ^2^School of Veterinary Medicine and Science, University of Nottingham, Sutton Bonington, United Kingdom; ^3^Department of Clinical Sciences, College of Veterinary Medicine, Cornell University, Ithaca, NY, United States

**Keywords:** canine hip dysplasia, genomic selection, labrador retrievers, genomic best linear unbiased prediction, joint reference population

## Abstract

Canine hip dysplasia, a debilitating orthopedic disorder that leads to osteoarthritis and cartilage degeneration, is common in several large-sized dog breeds and shows moderate heritability suggesting that selection can reduce prevalence. Estimating genomic breeding values require large reference populations, which are expensive to genotype for development of genomic prediction tools. Combining datasets from different countries could be an option to help build larger reference datasets without incurring extra genotyping costs. Our objective was to evaluate genomic prediction based on a combination of UK and US datasets of genotyped dogs with records of Norberg angle scores, related to canine hip dysplasia. Prediction accuracies using a single population were 0.179 and 0.290 for 1,179 and 242 UK and US Labrador Retrievers, respectively. Prediction accuracies changed to 0.189 and 0.260, with an increased bias of genomic breeding values when using a joint training set (biased upwards for the US population and downwards for the UK population). Our results show that in this study of canine hip dysplasia, little or no benefit was gained from using a joint training set as compared to using a single population as training set. We attribute this to differences in the genetic background of the two populations as well as the small sample size of the US dataset.

## Introduction

Canine hip dysplasia results from malformation of the coxo-femoral joint, which leads to hip laxity and often results in hip joint degeneration, painful arthritis, and lameness (Lewis et al., 2010a; Comhaire, 2014). Although surgical intervention can improve a dog's condition, the disorder cannot be cured and is a major health concern of dog owners, breeders, and organizations. It has been shown to have a heritable genetic basis (0.30–0.37; Lewis et al., 2013; Sánchez-Molano et al., 2015) and may thus be target for selection in order to reduce its prevalence.

In many countries, dogs are routinely evaluated for canine hip dysplasia on the basis of radiographs. From the radiograph, the Norberg angle on each hip can be measured, which reflects the laxity of the hip joint, although not perfectly (Dennis, 2012; Gaspar et al., 2016). In the British Veterinary Association (BVA)/Kennel Club (KC) Hip Dysplasia Scheme, a scale of 0–6 is used to categorize nine different components including the Norberg angle, where a healthy, unaffected hip (Norberg angle >105°) receives a score of 0. Scores increase with the severity of hip dysplasia, with the most severe score 6 (Norberg angle <79°) corresponding to the most extreme joint laxity (Dennis, 2012). Although quantitative measurements of Norberg angles contain more information than scores and are preferable for genetic evaluation (Woolliams et al., 2011), the latter are often used in aggregate scores with other hip traits which rely on qualitative scoring (Lewis et al., 2010b).

Breeding programs against canine hip dysplasia based on phenotypic thresholds and/or pedigrees have had only moderate success (Malm et al., 2008; Lewis et al., 2010a; Hou et al., 2013; Oberbauer et al., 2017). Genomic selection, which has been highly successful in dairy cattle (Hayes et al., 2009) and is being introduced into other livestock species (Cleveland and Hickey, 2013), has been suggested as a means for improved breeding against canine hip dysplasia (Malm et al., 2008; Guo et al., 2011; Woolliams et al., 2011). Sánchez-Molano et al. (2015) found that the prediction accuracies of genomic selection methods for Norberg angle, and related hip scores, were generally better than pedigree-based prediction in a study of Labrador Retrievers even with a reference set of fewer than 1,200 dogs.

The quality of genomic prediction depends on the size, structure, and relationship of the reference set, which are influenced by the effective population size of the reference population (Daetwyler et al., 2010). The UK population of Labrador Retrievers has an effective population size of N_E_ = 81.75–200 (Lewis et al., 2015; Wang et al., 2016), similar to that of cattle breeds such as Holsteins under intense selection (N_E_≈100) (Schöpke and Swalve, 2016). Where agricultural systems, such as those for cattle, have appropriate infrastructures to record and obtain large reference sets, dog breeding programs do not, and must rely on voluntary programs such as the BVA/KC Hip Dysplasia Scheme. Increasing the reference set by adding other datasets from related breeds or other countries is an option that has been investigated in cattle (Lund et al., 2014; Schöpke and Swalve, 2016) for improving the quality of genomic prediction. In these cases, when combining data from a large breed with data from a small breed, the prediction accuracy was improved for the small breed, while it was suggested that the larger breed might benefit from “a stronger persistence of the accuracy over generations” (Lund et al., 2014). Thus, one option for genomic selection against hip dysplasia is to explore the possibility of using a joint cross-country reference set.

In this study, we combine genotypic and phenotypic data from 1,179 UK Labrador Retrievers (Sánchez-Molano et al., 2014, 2015) and 242 US Labrador Retrievers (Hayward et al., 2016a,b). The aim of this study was to evaluate benefits of using a joint reference set of the two Labrador Retriever populations to predict Norberg angle scores to assess canine hip dysplasia.

## Materials and methods

### Datasets

The data in this study consists of two distinct datasets. The first dataset (“UK dataset”) is from our previous research by Sánchez-Molano et al. (2014, 2015) and dogs from this dataset will be referred to as “UK dogs.” The second dataset (“Cornell dataset”) is from the study of Hayward et al. (2016a) and dogs of this dataset will be referred to as “Cornell dogs.” The two datasets were also combined into one, referred to as “Joint.” An overview of the dataset is summarized in Table 1.

**Table 1:** Overview of dataset and phenotype.

**Origin**	**Sex**	**No**.	**NA**	**NA score**	**NA score ≤ 1(%)**	**NA score ≤ 2(%)**
Cornell	Male	116	104 ± 8.56	0.96 ± 1.47	78	88
	Female	126	103 ± 9.02	1.02 ± 1.60	79	87
UK	Male	293	-	0.95 ± 1.34	78	88
	Female	885	-	1.03 ± 1.24	71	87

*±, Standard deviation. No., Number of dogs; NA, Norberg angle*.

#### Ethics approval and consent to participate

This study did not conduct new procedures for collecting genotype or phenotype information. For full discussion of data collection, we refer to the original studies of Sánchez-Molano et al. (2014, 2015) and Hayward et al. (2016a). For the UK dataset (Sánchez-Molano et al., 2014, 2015), approval for buccal swab sampling of dogs was provided by the University of Edinburgh, Royal (Dick) School of Veterinary Sciences, Veterinary Ethical Review Committee and consent was provided by dog owners via completion of a questionnaire. For the Cornell dataset (Hayward et al., 2016a), blood samples were collected in accordance with the protocol approved by the Institutional Animal Care and Use Committee of Cornell University.

#### UK dataset

**Figure 1:**
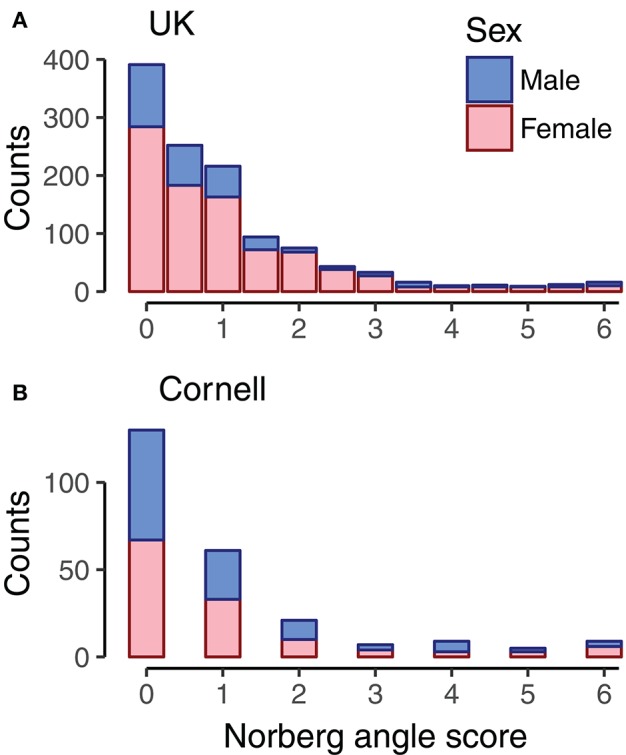
Histogram of Norberg angle scores in datasets. For UK dogs (**A**, *n* = 1,178), the Norberg angle score is the average of the Norberg angle score of left and right hip. For Cornell dogs (**B**, *n* = 242), the Norberg angle score is the score associated with the average Norberg angle of left and right hip. Bars are color coded by sex of dogs.

Genomic data from 1,179 Labradors Retrievers of both sexes were from Sánchez-Molano et al. (2014, 2015); among these Labradors, 1,178 had Norberg angles scored for left and right hip, and all dogs were <5 years old. Dogs were evaluated for hip dysplasia based on radiographs according to the UK scoring method (Willis, 1997; Sánchez-Molano et al., 2014), which evaluates nine components, including Norberg angle scores. The Norberg angle scores are whole integers in the range of 0 for a good hip to 6 as the most severe score for hip dysplasia (Dennis, 2012). The Pearson correlation between the Norberg angle scores of left and right hip in the current dataset was 0.60. For combining with the Cornell dataset, the average Norberg angle score of left and right hip was used. The majority of dogs had Norberg angle scores of 2 or below, with the remaining scores roughly equally spread on Norberg angle scores 2.5 to 6, as seen in Figure 1A. Due to using the average Norberg angle score, scores were multiples of 0.5.

Genotypes were obtained from buccal swabs and genotyped using the Illumina Canine High Density Beadchip. A total of 106,282 single nucleotide polymorphism (SNPs) remained after quality control procedures for low call rate, low reproducibility, low or confounded signal, significant deviations from Hardy-Weinberg equilibrium, and removal of SNPs with minor allele frequency (MAF) < 0.01 (Sánchez-Molano et al., 2014, 2015).

#### Cornell dataset

Data was downloaded 18th May 2016 from the Dryad repository (Hayward et al., 2016b) containing genotype and phenotype data. The canine hip dysplasia phenotype provided in this dataset was the mean Norberg angle of right and left hip for 242 Labrador Retrievers of both sexes. The dataset contained records that were restricted to Norberg angles above 75° and dogs more than 5 months of age to reduce outlier effects (Hayward et al., 2016a). We converted the mean Norberg angles assessed by Cornell to the UK scheme Norberg angle scores in accordance with Dennis (2012). Correlations between Norberg angles and Norberg angle scores was −0.97. The distribution of Norberg angle scores of the Cornell dogs (Figure 1B) was similar to that of the UK dogs (Figure 1A).

Genotyping was performed on blood samples using the Illumina CanineHD array with an additional 12,143 custom SNPs. Quality control was previously described by Hayward et al. (2016a) and the downloaded dataset contained 160,727 post-quality control SNPs. The current study carried out no further quality control.

#### Joint dataset

Data of the 242 Cornell Labrador Retrievers were combined with the data of the 1,179 Labradors from the UK dataset. Genotypes were merged using the merge function of PLINK v1.90b1g (Chang et al., 2015). All SNP positions were defined on the canFam3.1 assembly (Hoeppner et al., 2014). 105,848 SNPs were in common between the two datasets. SNPs defined in the UK dataset but not the Cornell dataset, and vice versa, was due to quality control performed in separately in each dataset and the addition of custom SNPs to the Cornell dataset. Of the SNPs in common, 104,430 had a minor allele frequency above 0.01 in the joint dataset and these were brought forward for all further analyses.

#### Relationships

We estimated relationships in the joint dataset by computing a genomic relationship matrix as per VanRaden's Method 1 (VanRaden, 2008); $G=ZZ^{\prime }/2\sum p_{i}(1-p_{i})$ where **Z** was the centered genotype matrix, and *p*_*i*_ are the allele frequencies estimated from the joint dataset. The average diagonal value was 1.019 (sd: 0.053) and 1.137 (sd: 0.101) for UK and Cornell dogs, respectively. The average off-diagonal value between UK dogs was 0.003 (sd: 0.077), between Cornell dogs 0.083 (sd: 0.107), and between UK and Cornell dogs −0.018 (sd: 0.050). The average F_ST_ between the two populations was estimated as 0.03 with PLINK v1.90b1g (Chang et al., 2015), consistent with intra-breed values for other dog populations (Quignon et al., 2007). Correlation between linkage disequilibrium of the two populations was estimated as 0.86, cf. Zhou et al. (2013).

### Genomic prediction accuracy

The prediction accuracy was evaluated by using a genomic best linear unbiased prediction (GBLUP) model, as follows:

(1) y=Wα+u+e

where **y** is the vector of Norberg angle scores, **W** is a matrix of covariates with the **α** vector of associated fixed effects including intercept, $\text{u}\;\sim \;MVN\left(0,\text{G}\sigma_{u}^{2}\right)$ is a vector of random genomic effects and $\text{e}\;\sim \;MVN\left(0,\text{I}\sigma_{e}^{2}\right)$ is a vector of residual errors. The genomic relationship matrix was calculated as described above, with an added small value (0.01) to the diagonal to allow inversion. The intercept, sex, and origin of the dogs (UK, Cornell) were used as covariates.

The Average-Information Restricted Maximum Likelihood (AI-REML) algorithm (Madsen et al., 1994; Johnson and Thompson, 1995), as implemented in DMU v. 5.1 (Madsen and Jensen, 2000), was used to fit the model to a subset of the data (training set), estimate variance components, and predict genomic effects in a separate data subset (validation set), which we henceforth refer to as predicted scores. We define convergence of the AI-REML algorithm based on the change of variance components, |θ^(*t*+1)^−θ^(*t*)^| < 10^−5^, where θ^(*t*)^ is the vector of normalized variance components estimated at step *t* (Jensen et al., 1997).

The prediction accuracies were calculated as the Pearson correlation (ρ) and mean-squared-error (MSE) between the true Norberg angle scores and predicted scores, and the slope of the linear regression of the observed Norberg angle scores onto the predicted scores (bias or inflation). A good prediction has a large ρ, small MSE, and a slope close to 1. A slope closer to 1 indicates a smaller prediction bias, with slopes >1 (<1) indicating under- (over-) estimation.

The heritability was calculated as $h^{2}=\sigma_{g}^{2}/\left(\sigma_{g}^{2}+\sigma_{e}^{2}\right)$. Standard errors (se) of the heritability estimates were calculated as

$$\begin{array}{l} \text{s}.\text{e}.\left(h^{2}\right)=\surd [{\left(\frac{\partial h^{2}}{\partial \sigma_{g}^{2}}\right)}^{2}{\left(se_{g}\right)}^{2}+{\left(\frac{\partial h^{2}}{\partial \sigma_{e}^{2}}\right)}^{2}{\left(se_{e}\right)}^{2}\\ \;+2\left(\frac{\partial h^{2}}{\partial \sigma_{g}^{2}}\right)\left(\frac{\partial h^{2}}{\partial \sigma_{e}^{2}}\right)\rho_{g,e}\;se_{g}\;se_{e}\;] \end{array}$$

with partial derivatives given as $\frac{\partial h^{2}}{\partial \sigma_{g}^{2}}=\frac{\sigma_{e}^{2}}{{\left(\sigma_{g}^{2}+\sigma_{e}^{2}\right)}^{2}}$, $\frac{\partial h^{2}}{\partial \sigma_{e}^{2}}=\frac{-\sigma_{g}^{2}}{{\left(\sigma_{g}^{2}+\sigma_{e}^{2}\right)}^{2}}$, and $\rho_{g,e}se_{g}se_{e}=\mathrm{cov}\left({\hat{\sigma }}_{g}^{2},{\hat{\sigma }}_{e}^{2}\right)$.

#### Prediction accuracy using 5-fold cross-validation

**Figure 2:**
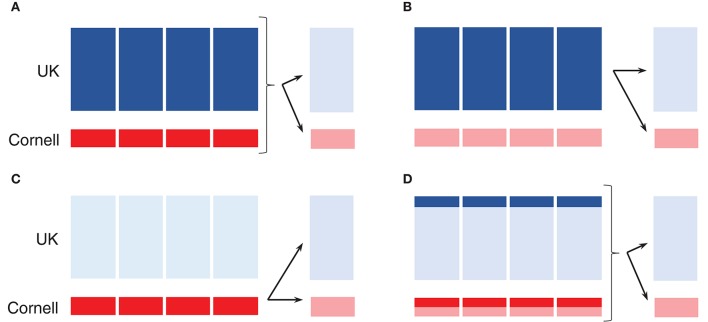
Schematic of 5-fold cross-validation using single origin or joint training set. **(A–D)** Dark shades displays training set, while arrows indicate prediction in the validation set in the disjoint group to the right. **(A)** Joint training set. **(B)** UK dogs as training set. **(C)** Cornell dogs as training set. **(D)** Subset of joint training set.

A 5-fold cross-validation scheme was used to estimate prediction accuracies. The UK dogs were randomly split into 5 groups (235–236 dogs per group), and the Cornell dogs were randomly split into 5 groups (48–49 dogs per group). Following this division, groups were defined as training (4 of the 5 groups) and validation (last of 5 groups) sets in three different ways: both UK and Cornell dogs as the training set (Figures 2A,D), only UK dogs as the training set (Figure 2B), or only Cornell dogs as the training set (Figure 2C). Prediction accuracies were then calculated in the remaining (validation) UK group and Cornell group, as indicated by arrows in Figures 2A–D. Reported prediction accuracies are averages across each 5-fold cross-validation. The 5-fold cross-validations were replicated 10 times, each time randomly splitting the dogs into 5 groups, giving an overall prediction accuracy average across the 10 replicates. For investigating the effect of increasing training set size, the above-mentioned 5-fold cross-validations were also performed on subsets of UK and Cornell dogs (Figure 2D). For these training sets, an equal number of dogs was sampled from each group to ensure a balanced setup.

#### Prediction accuracy between groups defined by principal component analysis

**Figure 3:**
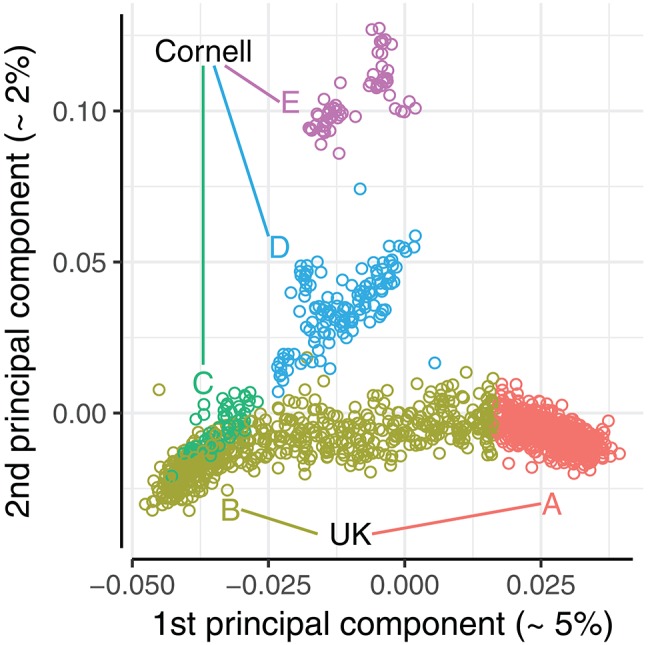
Plot of 1st and 2nd principal components of the joint dataset. Principal component analysis conducted with PLINK v1.90b1g (Chang et al., 2015). Dogs were split into 5 principal component groups (A-E) by visual inspection, and color coded accordingly.

A principal component analysis (PCA) was conducted to evaluate how the population structure of the combined dataset could affect the prediction accuracy. A PCA of the joint dataset was performed using PLINK v1.90b1g (Chang et al., 2015). The first two principal components explained 5.4 and 2.3% of the total variance, with remaining principal components each explaining <1%. The UK dogs were primarily stratified along the first principal component, as seen in the bottom band of Figure 3, while the Cornell dogs were stratified along both first and second principal components. The stratification of UK dogs corresponds roughly to the dogs' working class, i.e., with “gun dogs” primarily with positive values of PC1 (Group A, Figure 3) and showdogs primarily with negative values, co-clustering with a group of Cornell dogs (Group B and C, Figure 3). Pets were distributed across the entirety of the PC1 axis. The stratification of Cornell dogs was also found to be related to the provenance of the dogs: the group co-clustering with UK dogs (Group C, Figure 3) was primarily composed of dogs seen in the Cornell veterinary clinic, while dogs with high values of PC2 (Group E, Figure 3) were primarily from a closed colony. The cluster with remaining Cornell dogs (Group D, Figure 3) was comprised of both clinic and colony dogs.

We defined 5 PCA groups based on the first and second principal components, highlighted in Figure 3. The UK dogs were divided into two groups A and B of equal size (*n* = 589) by ranking the dogs by their first principal component values. The Cornell dogs were divided into three groups; group “C” comprises 54 dogs that appear to be genetically similar to the UK dogs, while groups “D” and “E” comprise 128 and 60 dogs, respectively, that cluster together. The Norberg angle scores in each group are summarized in Supplementary Figure 1. The mean genomic relationship between UK groups A and B was −0.047; the mean genomic relationships between Cornell groups were 0.033, 0.008, and 0.08 (C-D, C-E, and D-E, respectively).

The PCA groups defined by the principal component analysis were also used for defining training and validation sets, in this case with no cross-validation. Both single groups and different combinations of the PCA groups were used as training sets. Prediction accuracies were calculated separately for each remaining group not used in the training set.

#### Preselecting SNPs with genome wide association analysis for prediction accuracy

Prediction accuracies were estimated using subsets of SNPs, corresponding to 1, 5, 10, …, 98, 100% of all SNPs in the merged dataset. SNPs were selected either at random (“random SNPs”) or as the leading SNPs when ranked by the statistical significance of association in a genome-wide association (GWA) analysis (“pre-selected SNPs”). The GWA analyses were performed as in Sánchez-Molano et al. (2014), using the software GEMMA (Zhou and Stephens, 2012), described below for completeness.

Briefly, the GWA analysis was performed using the linear mixed model

(2) $$y=W\alpha +x_{\text{i}}\beta_{\text{i}}+u+e$$

which is similar to the GBLUP, but with the addition of the estimation of each SNP effect $\beta_{i}\;\sim \;N\left(0,\sigma_{\beta }^{2}\right)$. The statistical significance of the association was estimated using the Wald's test ($t_{i}={\hat{\beta }}_{i}/se\left({\hat{\beta }}_{i}\right)$), which is assumed to be χ^2^ distributed with 1 degree of freedom. Based on this test, a *p*-value for each SNP-trait association was calculated.

For the pre-selected SNPs, a 5-fold cross-validation scheme was used to estimate *p*-values and prediction accuracies. The 5-fold cross-validation is as described above but with no replication. For each iteration of the cross-validation, the same training set was used for the GWA analysis to rank SNPs and to train the GBLUP model to avoid inflating the prediction accuracy (Wray et al., 2013). The genomic relationship matrix for the GBLUP model was constructed on the subset of SNPs. We emphasize that the SNP effects estimated in the GWA analysis were not used for predicting genetic values, but solely for ranking the SNPs. Instead, SNP effects were estimated jointly in the GBLUP model (unlike the GWA analysis, which estimates them independently).

For randomly selected SNPs, the same approach was used, except the SNPs were selected at random from across the genome. 20 replicates of each subset size of SNPs were performed.

## Results

Genomic heritabilities, calculated using AI-REML estimated variance components without 5-fold cross-validation, were 0.24 (se: 0.15) when calculated using only UK dogs, 0.73 (se: 0.21) when calculated using only Cornell dogs, and 0.28 (se: 0.14) when calculated using the joint training set. The heritabilities estimated as part of the 5-fold cross-validations had average values of 0.24 (UK dogs, range: 0.19–0.32), 0.66 (Cornell dogs, range: 0.36–0.99), and 0.28 (joint, range: 0.20–0.36). The genetic correlation of Norberg angle scores between the two populations was estimated as 0.52 (se: 0.28), as estimated using a bivariate GBLUP model.

### Predictive ability

Using the joint training set with both UK and Cornell dogs, validation in the Cornell dogs achieved a higher correlation (0.26) than the UK dogs (0.19) (Table 2). Converting these to accuracy of selection ($\rho /\sqrt{\;}h^{2}$), they are similar in size, 0.36 and 0.34, for UK and Cornell respectively.

**Table 2:** Overall prediction accuracies (correlation, MSE, and slope of linear regression) of 10 replicates of 5-fold cross-validations with different training sets.

	**Training**		
	**UK ($\bar{n}$ = 943)**	**Joint ($\bar{n}$ = 1,137)**	**Cornell ($\bar{n}$ = 194)**
**CORRELATION**			
UK	0.18 (0.16–0.21)	0.19 (0.17–0.20)	0.06 (0.05–0.07)
Cornell	0.14 (0.10–0.18)	0.26 (0.21–0.31)	0.29 (0.23–0.33)
**MSE**			
UK	1.55 (1.53–1.57)	1.54 (1.52–1.56)	1.67 (1.65–1.70)
Cornell	2.24 (2.21–2.27)	2.16 (2.08–2.22)	2.13 (2.05–2.24)
**SLOPE**			
UK	0.96 (0.84–1.12)	0.88 (0.80–0.97)	0.25 (0.17–0.33)
Cornell	1.18 (0.89–1.56)	1.19 (1.08–1.38)	1.00 (0.72–1.20)

*Figures in parentheses are minimum and maximum of the 10 replicates of 5-fold cross-validations. $\bar{n}$ refers of average size of training set*.

Using only UK dogs for the training set (second column of Table 2) the correlation for UK dogs was similar (0.18) to that of the joint training set, while the correlation for the Cornell dogs dropped substantially to 0.14. Using only Cornell dogs as training set, the correlation for the Cornell dogs increased to 0.29, while the correlation for the UK dogs dropped to 0.061.

The mean-squared-errors were similar across the three training sets (UK, Cornell, and joint) and substantially higher for the Cornell dogs than the UK dogs (Table 2).

The slope of a linear regression of the observed Norberg angle scores onto the predicted scores were all except one close to 1, i.e., there was little bias. Using Cornell dogs to predict UK dogs, the slope was 0.25 (Table 2), i.e., the bias increased. The smallest bias was seen when using Cornell dogs to predict Cornell dogs, or UK dogs to predict UK dogs.

#### Effect of increasing training set size

**Figure 4:**
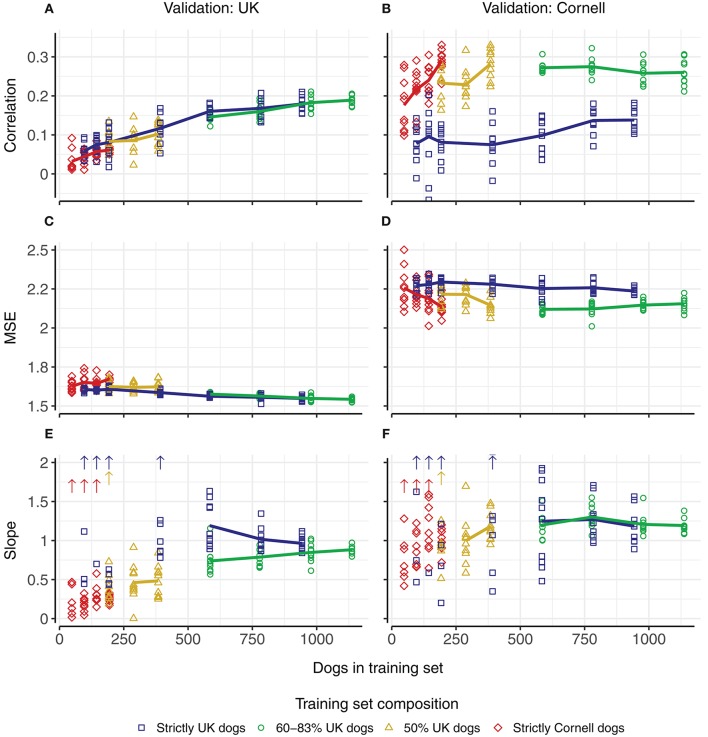
Predictive ability in UK and Cornell dogs with changing training set sizes and compositions. Columns correspond to predictions in UK dogs (left) and Cornell dogs (right). Rows correspond to correlation **(A,B)** between observed Norberg angle scores and predicted scores, mean-squared-errors **(C,D)**, and slope of regression **(E,F)** of observed Norberg angle scores onto predicted scores. Composition of training set is displayed by color. Each point corresponds to the average of a single 5-fold cross-validation (10 replicates), with solid lines connecting the overall average of the replicates. Upward pointing arrows denote where slope estimates exceed the displayed scale.

Increasing the number of dogs in the training sets increased the correlations (Figures 4A,B). Using only UK dogs for the training set (blue squares, Figure 4A), the overall average correlation for the UK dogs showed a steady increase from 0.06 (–0.03–0.09) using 96 dogs to 0.18 (0.16–0.21) using 943 or 944 dogs (blue, Figure 4A). Using only Cornell dogs for the training sets (red diamonds, Figure 4B), the average correlations for the Cornell dogs also showed an increase from 0.18 (0.10–0.28), using 48 dogs, to 0.29 (0.23–0.33), using 194 dogs (red, in Figure 4B).

Using a joint training set with equal proportions of UK and Cornell dogs (yellow triangles), increasing the number of dogs in the training set increased the correlations for both UK dogs (Figure 4A) and Cornell dogs (Figure 4B). When using all Cornell dogs (except dogs in the validation set) for the training sets and an increasing number of UK dogs (green circles), the increase in correlations for UK dogs (Figure 4A) matched the increase when using only UK dogs as training set (blue squares, Figure 4A). The correlations for Cornell dogs were not improved compared to using only Cornell dogs as the training set (green circles vs. red triangles, Figure 4B).

The mean-squared-errors of the prediction (Figures 4C,D) were affected minimally by increasing the size of the training set. The bias of the prediction (slope of the linear regression of the observed Norberg angle scores onto the predicted scores, where a slope closer to 1 indicates a smaller bias) is shown in Figures 4E,F. There are two things to note regarding the bias; training set sizes above 500 have slopes close to 1, while slopes for training set sizes below 500 vary widely, and with some estimates (substantially) >2, as indicated by the arrows. For training set sizes above 500, using a joint training set to predict Cornell dogs displays smaller variation than using only UK dogs. For predicting UK dogs, the slope converges toward 1 with increasing training set size.

For the smaller training sets, the correlations for the UK dogs are close to zero. This is largely coupled to estimates of slopes of regressions that are either close to zero or are orders of magnitude >2, as may be expected with zero correlation. The slope estimates for the Cornell dogs as predicted by Cornell dogs also display some cases with very large estimates, but these are not coupled with correlations close to zero.

#### Effect of composition of training set

Increasing the training set size by adding Cornell dogs to a training set of UK dogs (green and yellow lines, Figure 4A), had a similar positive effect on correlations as adding additional UK dogs (blue line, Figure 4A) but the slope of regressions deviated more from 1 (i.e., larger biases) compared to the addition of UK dogs (Figure 4E, blue vs. green line).

Increasing the training set size by adding UK dogs to a training set of Cornell dogs (green and yellow lines, Figure 4B), did not, however, increase the correlation for the Cornell dogs compared to the use of only Cornell dogs (red line, Figure 4B). The slope of the regressions, when ignoring extreme values, deviated more from 1 when using a joint training set rather than only Cornell dogs for predictions in Cornell dogs (Figure 4F).

#### Predictive ability between PCA groups

**Figure 5:**
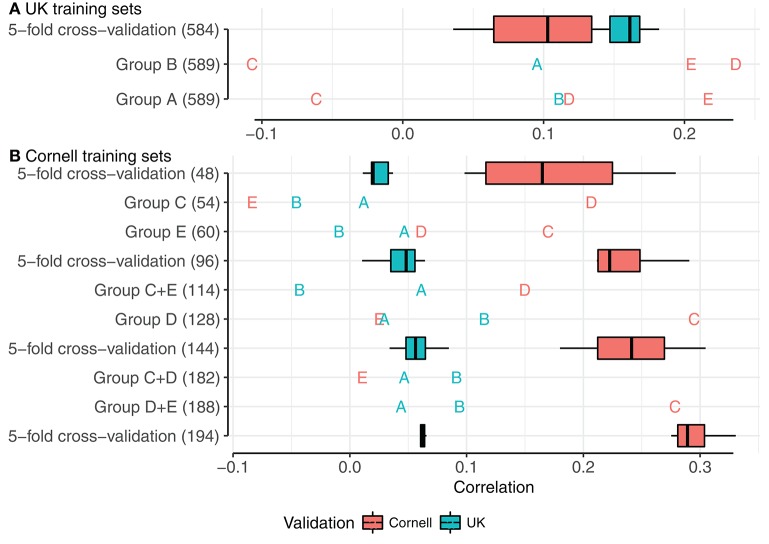
Correlations using PCA groups as training sets, with relevant 5-fold cross-validations of mixed groups. **(A,B)** show training sets of UK dogs and Cornell dogs, respectively. Boxplots and annotations are colored red for correlations in Cornell dogs and blue for correlations in UK dogs. Letters display PCA group as validation set.

To investigate how the genetic distances between dogs influenced prediction accuracy, we divided the joint dataset into five PCA groups, as defined by the principal component analysis (Figure 3). We used each of these PCA groups as training set and validated against remaining PCA groups. The results are summarized in Figure 5, with box-plots corresponding to correlations of the 5-fold cross-validations from the full datasets (Figure 4).

Correlations using UK groups A or B as the training set, each comprising 589 dogs, are summarized beneath the box-plot 5-fold cross-validations of a mixed group of 584 UK dogs (Figure 5A). Using UK group A to predict UK group B, and vice versa, resulted in similar correlations (≈0.1), but both correlations were lower than achieved by the 5-fold cross-validations of the mixed group (0.18). Using groups A or B as training sets to predict the Cornell groups gave variable results, with highest correlations for groups D and E (>0.2) and lowest for group C (<−0.05), despite the PCA clustering seen between Cornell group C and UK group B.

Using Cornell groups C, D, and E as training sets is summarized Figure 5B. The sizes of these training sets were noted above for producing inferior predictions in UK dogs or highly variable predictions in Cornell dogs. Using the small groups C and E separately produced correlations for UK groups A and B close to zero and resulted in estimated heritabilities of 1 (with very flat likelihood profiles, indicating very low power for estimation). Group D, which was slightly larger than C and E combined, produced higher correlations for group B (≈0.12) than the 5-fold cross-validations (≈0.05), and adding either group C or E had detrimental effects on the correlations of group B (<0.1).

#### Effect of pre-selecting SNPs

**Figure 6:**
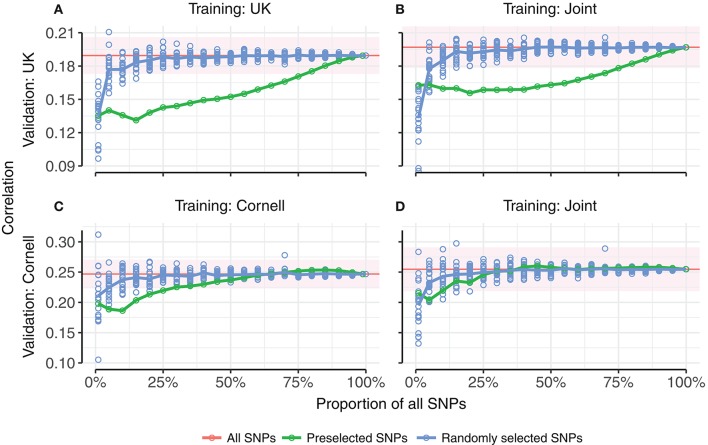
Prediction accuracies as a function of proportion of SNPs used for prediction. **(A,B)** show correlations of predictions in UK dogs using UK dogs or joint training set, respectively. **(C,D)** show correlations of predictions in Cornell dogs using Cornell dogs or joint training set, respectively. Random selection of subsets of SNPs for genomic prediction (blue points, 20 replicates per subset size) approaches the correlations for all SNPs (red line) more quickly than pre-selected subsets of SNPs ranked by genome-wide association (green points). Points are averages of 5-fold cross-validations. The red horizontal line indicates the average correlation of 5-fold cross-validation using all SNPs (20 replicates), with standard error of average indicated by the red ribbon.

Random selection of SNPs approached the same correlation as all SNPs more quickly than pre-selecting SNPs based on strength of phenotypic association (Figure 6). It can be seen that in all four tested combinations of training set (UK, Cornell or joint) and validation set (UK or Cornell), fewer than 20% of the random SNPs achieved the same correlations as using all SNPs, whereas more than 25% of pre-selected SNPs (90% for the UK validation set) were required to reach the level of all SNPs. The only case where pre-selected SNPs performed (marginally) better than random SNPs was for a very small subset (1%) of SNPs, when using the joint training set. The difference between pre-selected and random SNPs was less pronounced for validation in Cornell dogs than UK dogs. The estimates using all SNPs (based on a single realization of the training and validation sets) are within the range of 5-fold cross-validations (Table 2).

Random SNPs performed better than pre-selected SNPs in terms of bias (Supplementary Figure 2), where even the smallest subset of random SNPs achieved the same slope of regression as using all SNPs.

**Figure 7:**
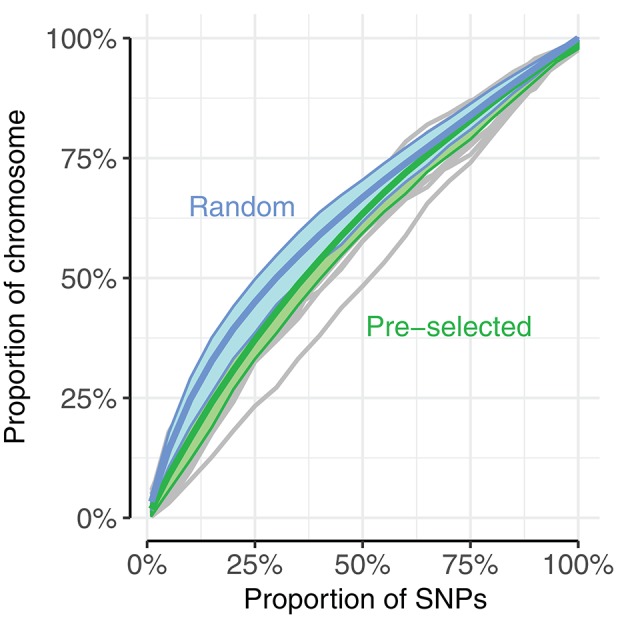
Randomly selecting SNPs (blue) resulted in higher proportion of chromosome coverage than pre-selecting SNPs (green). Genome coverage among the UK dogs was estimated as the sum of spanned haplotype blocks mapped by selected SNPs, compared to the total span of haplotype blocks. Blue, thick line corresponds to average of 20 replicates of randomly selected SNPs, with blue area displaying range between 10th and 90th percentile of coverage. Thin gray lines correspond to coverage by pre-selected SNPs of each chromosome (chromosome 35 showed unusually low coverage compared to the other chromosomes, seen in the bottom gray line); thick green lines display average of all chromosomes with 10th−90th percentile. SNPs were pre-selected according to a single subset of the 5-fold cross-validation.

There are various differences between the use of random and pre-selected SNPs in the context of genomic prediction. We explored one of these, differences in genome coverage, by calculating the proportion of the genome covered by SNP subsets of increasing size for the UK dataset. The proportion of the genome covered was estimated by calculating haplotype blocks using PLINK v1.90b1g (Chang et al., 2015), and summing the range of blocks with selected SNPs. The coverage by haplotype blocks is consistently larger when selecting random SNPs than pre-selected SNPs, up to approximately 60% of SNPs (Figure 7: blue line vs. green line). For example, randomly selecting 25% of the SNPs results in haplotype blocks covering 46% of the genome, while pre-selecting the same number results in haplotype blocks covering 37% of the genome.

## Discussion

In this study, we addressed the need for building training sets for genomic prediction of hip dysplasia in canine breeds. Genomic evaluation has transformed breeding structures of livestock in populations with similar effective populations sizes to Labrador Retrievers through delivering substantially greater accuracies of predicting breeding values in unphenotyped animals, e.g., at birth. Sánchez-Molano et al. (2015) showed that for hip dysplasia, genomic evaluation has the potential to accelerate the reduction of hip problems, directly through greater accuracy, and indirectly by increasing the selection intensity by making accurate evaluations available at birth. The success in livestock has however been built upon an infrastructure capable of generating large training sets for predictions, typically several thousand genotyped and phenotyped animals are required for robust benefits beyond pedigree-based predictions (e.g., Jenko et al., 2016), with the benefits continuing to accumulate beyond these sample numbers. Despite the potential benefits, this will be a challenge for pedigree dog populations in the absence of well-supported breed initiatives. One option to address this challenge would be to pool training sets across sub-populations and, as here, across countries. However, the results did not provide encouragement that this would be a fruitful way forward at present.

The use of Norberg angle as phenotype for this study was necessitated by the use of different scoring systems in the two countries. With larger datasets in both countries, multivariate approaches could be used to overcome this problem, whereby the best indices for the risk of hip dysplasia could be used within each country and then compared across countries. However, the relatively small datasets prompted the use of Norberg angles, which were available in both sets. This was a robust choice as it is moderately heritable (h^2^≈0.3), as is hip score (Lewis et al., 2010a,b), with a substantial genetic correlation with hip score, and a major determinant of the morphological indices explored by Lewis et al. (2010b), which were shown to be near-optimum predictors of genetic risk. Furthermore, a key underlying determinant of the utility of cross-country predictions will be the genetic relationships between the two populations, which will likely depend as much on their population structures and relationships as on the choice of traits. Nevertheless, the use of Norberg angle required obtaining a common measure of the trait, as UK scores were ordinal rather than nominal as in the Cornell dataset. In principle, transforming the phenotype from a nominal to an ordinal scale, as done here with Cornell's Norberg angles, can lower the resolution and impair the prediction. However, the phenotypic correlation between Norberg angles and derived scores for the Cornell data had a large magnitude (−0.97), indicating that this transformation to scores is not responsible for the low cross-country prediction accuracy observed here (see Supplementary Figure 3). A corollary to this observation is that although the ordinal scale used in the UK is an unnecessary coarsening of a quantitative measure, its use in BLUP evaluations (Lewis et al., 2010a) rather than the nominal angle may have little effect on the accuracies achieved.

The lack of benefit from combining UK and Cornell training sets was most evident when supplementing the Cornell training sets with data from UK dogs where the prediction accuracy fell. It would be expected that any potential for increasing accuracy by combining data would have been most evident in this case as adding UK dogs increased the training set for Cornell dogs six-fold. The small increase (0.18–0.19) in predicting phenotypes of UK dogs by adding Cornell data may well have been due to chance. Within countries, larger training sets did provide more accurate predictions for dogs from their corresponding populations, which is in accord with current literature (Goddard, 2009; Pszczola et al., 2012), and clearly points to the mixed origin within the training and validations sets as the underlying reason for the lack of benefit of combining datasets to increase training set sizes.

The failure of the addition of foreign dogs to improve correlations is most likely due to insufficient relationship between the datasets. Estimation of relationship by calculating a genetic relationship matrix also showed that the relationship between the UK and Cornell populations was generally low (−0.018) and the relationship between Cornell dogs (0.083) was generally higher than among UK dogs (0.003). These values may also explain the higher prediction correlations among Cornell dogs, as the greater relationship represents more mutual information and hence greater predictive ability per dog in the training set. In comparison to similar studies in livestock (Danish and Chinese Holstein cattle, Zhou et al., 2013) where the prediction improved from joint training sets, there was a higher level of consistency of the linkage disequilibrium in the two cattle populations (*r*_*LD*_ = 0.97 compared to 0.86 in ours). It is possible that specific sub-populations within the Cornell and UK datasets were sufficiently distinct to be responsible for the failure of the across-country training sets, however there was no evidence that removing any of the subsets would dramatically change the predictive outcomes. In a related study using multi-breed dairy cattle (Karoui et al., 2012), using multi-breed models did not increase accuracy of predicted values for fertility, a low heritability trait. This was explained by a low genetic correlation between the breeds for the trait, indicating that linkage disequilibrium between genetic markers and quantitative trait loci was not consistent between the breeds. In comparison, we found a greater genetic correlation between populations (0.53, se: 0.28), but as it was estimated with a large standard error and not significantly >0 (Wald's test, *p* = 0.06), this is not very informative. The use of pre-selected SNPs that are enriched in causative variants or markers very tightly linked to causative variants may be useful for capturing shared variation across distant populations (e.g., Porto-Neto et al., 2015). However, the studies of pre-selected SNPs conducted in this study were not encouraging, supporting the results of Sánchez-Molano et al. (2015), showing that for Labradors with the current state of genomic knowledge, the use of random SNP provided more accurate results. The total UK plus Cornell training set of 1,420 dogs used in this study is large in the context of canine genomics, albeit with 83% from UK, and prompts the conclusion that combining data across these countries is unlikely to be very effective in boosting prediction accuracies in the short to medium term. This places the emphasis on initiatives within countries and within sub-populations. One low-cost route to increase accuracies would be to use single step methods (Legarra et al., 2014), which combine the pedigree data and the genomic data in a single analysis, hence exploiting all the data in recording schemes to get maximum benefit from the phenotyped and genotyped individuals. A second route is to increase genomic training set sizes by developing affordable cost-efficient genotyping schemes and two strategies can be implemented to deliver such schemes. First, the use of imputation whereby cheaper low-density genotyping is used for the majority of the population but key individuals used widely in the population are genotyped at higher target density and software such as AlphaImpute (Hickey and Kranis, 2013; Antolín et al., 2017) is used to infer the missing genotypes; such strategies have been demonstrated to be effective in livestock populations, benefitting from their low N_e_. A second strategy is selective genotyping, following Jenko et al. (2016) who demonstrated in Guernsey Island cattle that more than 80% of the information could be captured by genotyping only the top 25% and the bottom 25% of the population.

We conclude that predicting Norberg angle scores in UK Labrador Retrievers using a joint training set of both UK and Cornell Labrador Retrievers was feasible, but the benefits were negligible compared to using only training sets of a single population, and the inclusion of UK dogs in the training set worsened predictions for Cornell dogs. Improving the prediction accuracy of Norberg angle scores requires large datasets that are closely related to the target populations, which was not the case with these two geographically distant countries. The way forward will thus be to increase datasets within groups of countries with well-connected pedigrees. We also found that very small training sets did not contain enough information for prediction and the use of SNPs preselected based on GWAS results did not improve prediction accuracy. The difference between the genetic backgrounds of the two populations may require more nuanced models than univariate genomic BLUP.

## Availability of data and material

The “Cornell” dataset analyzed during this study is available in the Dryad repository (https://doi.org/10.5061/dryad.266k4). The “UK” dataset of 1,179 Labrador Retrievers is available from the corresponding author on reasonable request.

## Author contributions

SE and PW conceived and designed the study. SE performed the data analysis and drafted the manuscript. JW and PW oversaw the analysis and helped to interpret the results and refine the manuscript. JH and RT helped to interpret the results and refine the manuscript. SB, DC, and ES-M contributed to data collection and management. All authors read and approved the final manuscript.

### Conflict of interest statement

The authors declare that the research was conducted in the absence of any commercial or financial relationships that could be construed as a potential conflict of interest.

## Supplementary material

Supplementary Figure 1 **(A–E)** Distribution of Norberg angle scores in PCA groups A–E.

Supplementary Figure 2 Few randomly selected SNPs are necessary to achieve same slope as using all SNPs. **(A,B)** show slopes of regression of observed Norberg angle scores onto predicted scores in UK dogs using UK dogs or joint training set, respectively. **(C,D)** show slopes of predictions in Cornell dogs using Cornell dogs or joint training set, respectively. Selecting SNPs by GWA requires all SNPs to achieve the same slope as using all SNPs. Points are averages of 5-fold cross-validations. The red horisontal line indicates the average correlation of 5-fold cross-validation using all SNPs (20 replicates), with standard error of average indicated by the red ribbon.

Supplementary Figure 3 Correlation between Norberg angle and Norberg angle scores **(top)** and distribution of Norberg angles **(bottom)** for Cornell dogs.
