# Supplementary figures and images for: Joint Genomic Prediction of Canine Hip Dysplasia in UK and US Labrador Retrievers
Source: Front Genet. 2018 Mar 28;9:101. doi: 10.3389/fgene.2018.00101 (PMC5883867; doi:10.3389/fgene.2018.00101)

Sex

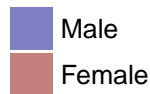

A – UK

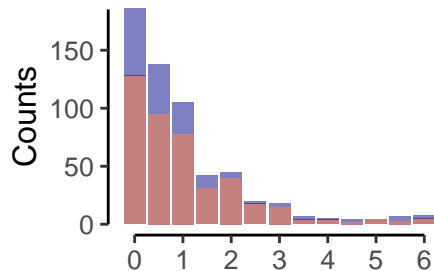

B – UK

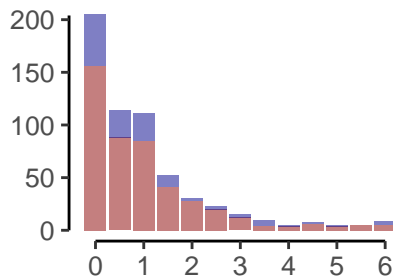

C – Cornell

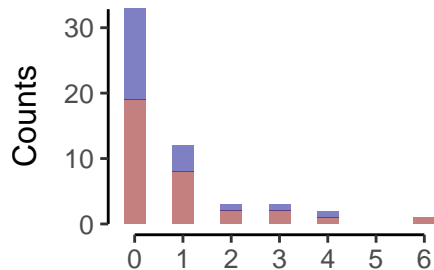

D – Cornell

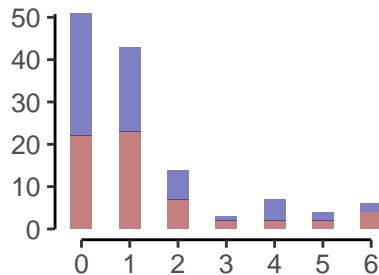

E – Cornell

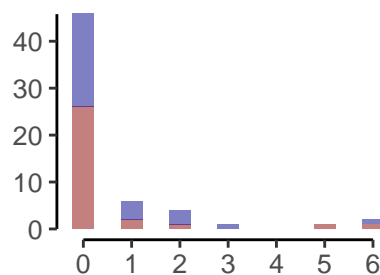

Norberg angle score

Supplement: Supplementary Figure 1 — (A–E) Distribution of Norberg angle scores in PCA groups A–E. [file Image1.PDF]

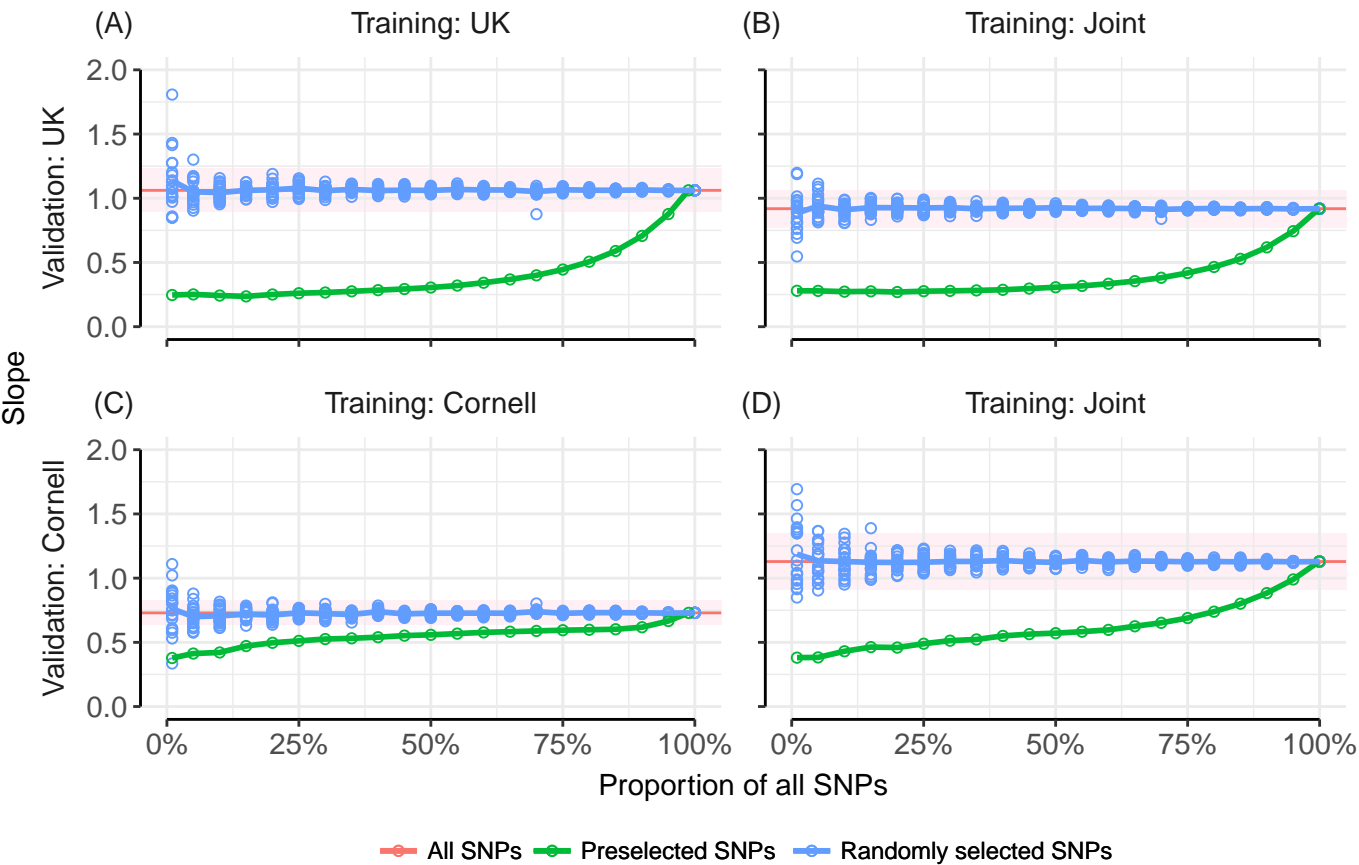

Supplement: Supplementary Figure 2 — Few randomly selected SNPs are necessary to achieve same slope as using all SNPs. (A,B) show slopes of regression of observed Norberg angle scores onto predicted scores in UK dogs using UK dogs or joint training set, respectively. (C,D) show slopes of predictions in Cornell dogs using Cornell dogs or joint training set, respectively. Selecting SNPs by GWA requires all SNPs to achieve the same slope as using all SNPs. Points are averages of 5-fold cross-validations. The red horisontal line indicates the average correlation of 5-fold cross-validation using all SNPs (20 replicates), with standard error of average indicated by the red ribbon. [file Image2.PDF]

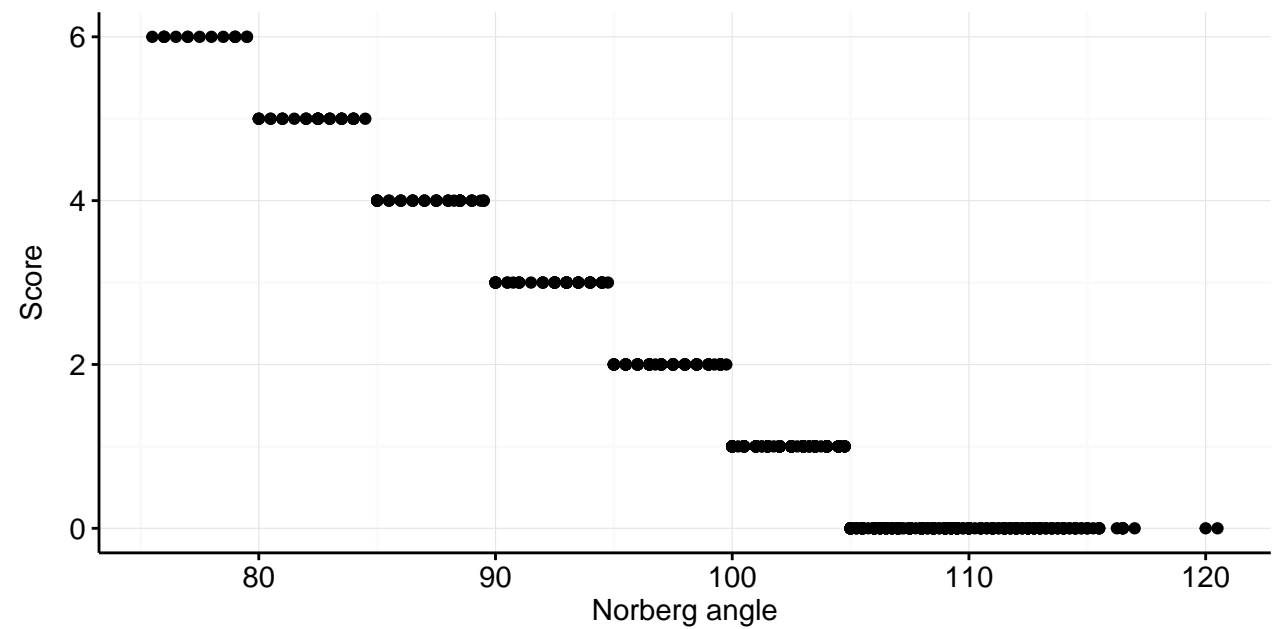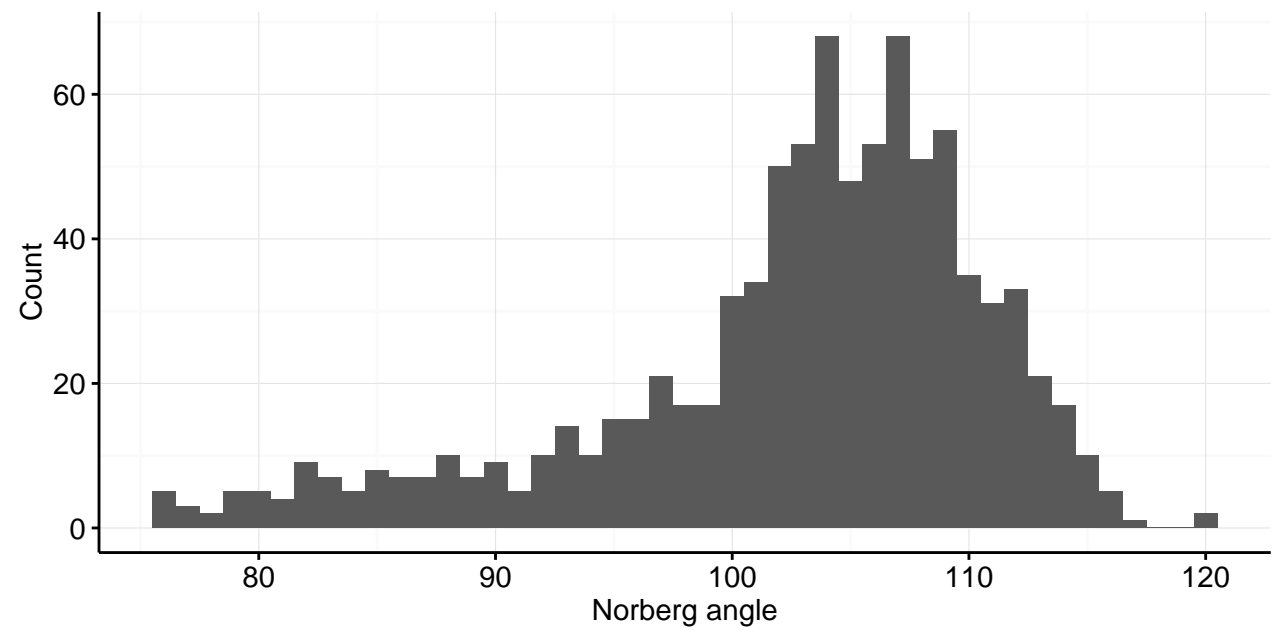

Supplement: Supplementary Figure 3 — Correlation between Norberg angle and Norberg angle scores (top) and distribution of Norberg angles (bottom) for Cornell dogs. [file Image3.PDF]
